# Prevalence and predictors of alcohol use among adult males in Ethiopia: multilevel analysis of Ethiopian Demographic and Health Survey 2016

**DOI:** 10.1186/s41182-020-00287-8

**Published:** 2020-12-07

**Authors:** Zemenu Tadesse Tessema, Tadele Amare Zeleke

**Affiliations:** 1 Department of Epidemiology and Biostatistics, Institute of Public Health, College of Medicine and Health Science, University of Gondar, Gondar, Ethiopia; 2 Department of Psychiatry School of Medicine, College of Medicine and Health Science, University of Gondar, Gondar, Ethiopia

**Keywords:** Predictor, Ethiopia, Multilevel, Alcohol use

## Abstract

**Background:**

Alcohol is a psychoactive substance that is widely consumed in the world. Alcohol use is one of the world’s leading risk factors for disease and disability. It affects individuals’ physical, mental, economic, and social issues. To our knowledge, there is limited study on alcohol consumption and associated factors. Therefore, this study aimed to determine the prevalence and predictors of alcohol use in Ethiopia by using the 2016 Ethiopian Demographic and Health Survey.

**Methods:**

This study was based on the most recent Ethiopian Demographic and Health Survey 2016. A total of 12,594 men at the age of 15 to 59 were included in this study. Considering the hierarchical nature of EDHS data, a multilevel logistic regression model was applied. The ICC, MOR, and the LR test were done to assess the presence of a significant clustering effect. Besides, deviance was used for model comparison since the models were nested models. Variables with a *p* value ≤ 0.2 in the bivariable analysis were considered for the multivariable analysis. In the multilevel logistic regression, the adjusted odds ratio (AOR) with 95% confidence interval (CI) was reported to declare the strength and significance of the association between the dependent variable and independent variables.

**Results:**

The prevalence of alcohol drinking in this study was 46.64% with a 95% CI of 45.00 to 47.00%. Age groups 30–44 (AOR = 1.30, 95% CI 1.08, 1.56) and 45–59 (AOR = 1.38, 95% CI 1.10, 1.74), Orthodox religion follower (AOR = 0.36, 95% CI 0.24, 0.55), media exposure (AOR = 1.67, 95% CI 1.41, 2.20), khat chewing (AOR = 3.08, 95% CI 2.54, 3.74), smoking (AOR = 2.18, 95% CI 1.71, 2.79), having no occupation (AOR = 0.34, 95% CI 0.22, 0.51), and region were the predictors of alcohol use in Ethiopia.

**Conclusions:**

Nearly half of the Ethiopian population reported alcohol use at least once in their lifetime. Old age, Orthodox religion followers, media exposure, khat chewing, smoking, and having no occupation were predictors of alcohol use in Ethiopia. Therefore, health education about the risk of alcohol used is highly recommended. In addition, khat chewing and smoking control mechanisms should be designed and given special attention. Advertising alcohol through media is better to be controlled. Job opportunities should also be created for those who have no occupation to mitigate alcohol use in Ethiopia.

## Introduction

Alcohol is a liquid that contains ethanol and the most predominant beverage worldwide [1]. Alcohol use is one of the world’s leading health risks that results in 2.5 million death each year [2]. It is also a causal factor in many diseases and a precursor to injury, violence, and cardiovascular diseases [3]. Worldwide, alcohol use is associated with maternal and child health problems, risky sexual behavior, unintended pregnancy, injury, and poisoning [1]. Alcohol use triggers a host of public health harms, from injury and death accompanied by excessive drinking to increased violence, crime, poverty, and other forms of social destabilization, financial, disease, and death [4–6].

Alcohol is one of the most used and misused substances in different societies [7]. Due to alcohol drinking, 3 million Canadians are at risk of acute illness [8]. Alcohol is widely consumed by more than half of the population in the Americas, Europe, and the Western Pacific [1]. In Europe, alcohol leads to ischemic cardiovascular disease and injury, and death [9]. In England, about 10 million people are drinking at a level of which increases their risk of health problems. In age 15 to 49, alcohol is the leading cause of ill health, early mortality, and disability and the fifth leading cause for ill health in all age groups [10].

In low- and middle-income countries, alcohol use disproportionately affects premature mortality and disability [11]. In African countries, alcohol consumption has a large impact on the burden of disease and mortality, and alcohol exposure is expected to increase in the next years [12]. In the African continent, alcohol industry involvement and investment are rising following a general strategy to increase demand, availability, and access to alcoholic beverages [13].

Studies had been reported that the lifetime prevalence of alcohol use in New Zealand was 95.0% [5], in India 49.7% [14], in Nigeria 57.9% [15], in Uganda 51.4% [16], in Nigeria 76.0% [17], in Kenya 10.8% [18], and in Sri Lanka 53.7% [19].

Contributing factors for alcohol use were male sex [15, 20–23], being Christian followers (51.6%) [22], smoking [20, 23, 24], age 20–29 [20], increasing age, poor social support [25], being age 30 and older, low level of education [26, 27], and being in lower socioeconomic groups [28].

Alcohol advertising and marketing are misleading the public in order to entice them to consume alcohol [21, 29]. As a result, alcohol consumption is common and widely acceptable across all categories of people [30].

In Gondar Ethiopia, the prevalence of ever alcohol use was 48.23% [22]; in Ethiopia, systematic review and meta-analysis, pooled current and lifetime alcohol use were 23.86% and 44.16% respectively [31]. In another community study conducted in Ambo, Ethiopia, the prevalence of alcohol use disorder was 27% [32]. Alcohol consumption in Ethiopia is a risk factor for infectious diseases (tuberculosis, lower respiratory infections, viral hepatitis, sexually transmitted diseases including HIV), non-communicable diseases (heart diseases, non-infectious liver diseases, cancer), and mental disorders (alcohol use disorders including depression) [2]. As far as we know, in Ethiopia, studies showed that alcohol is the risk for different diseases and injuries rather than showed cause and effect relationship. Even though the risk of alcohol use is known in Ethiopia, less emphasis is given to the prevention strategies and the management of hazardous alcohol drinkers and addicted individuals [7]. Today, in Ethiopia, alcohol advertisement is prohibited with proclamation No. 759/2012. Advertised liquor with more than 12% alcoholic content may not be disseminated through mass media. Any liquor outdoor advertisement may not be placed within 100-m radius of children care center, school, medical or historical institution, cinema, theatre, and stadiums [33]. However, the fact is that this is not implemented. To our knowledge, there is a limited study on alcohol consumption and associated risk factors. Therefore, this study aimed to determine the prevalence and predictors of alcohol use in Ethiopia using the 2016 Ethiopian Demographic and Health Survey. The finding is crucial for policymakers and health professionals for effective intervention.

## Methods

### Study area

**Fig. 1:**
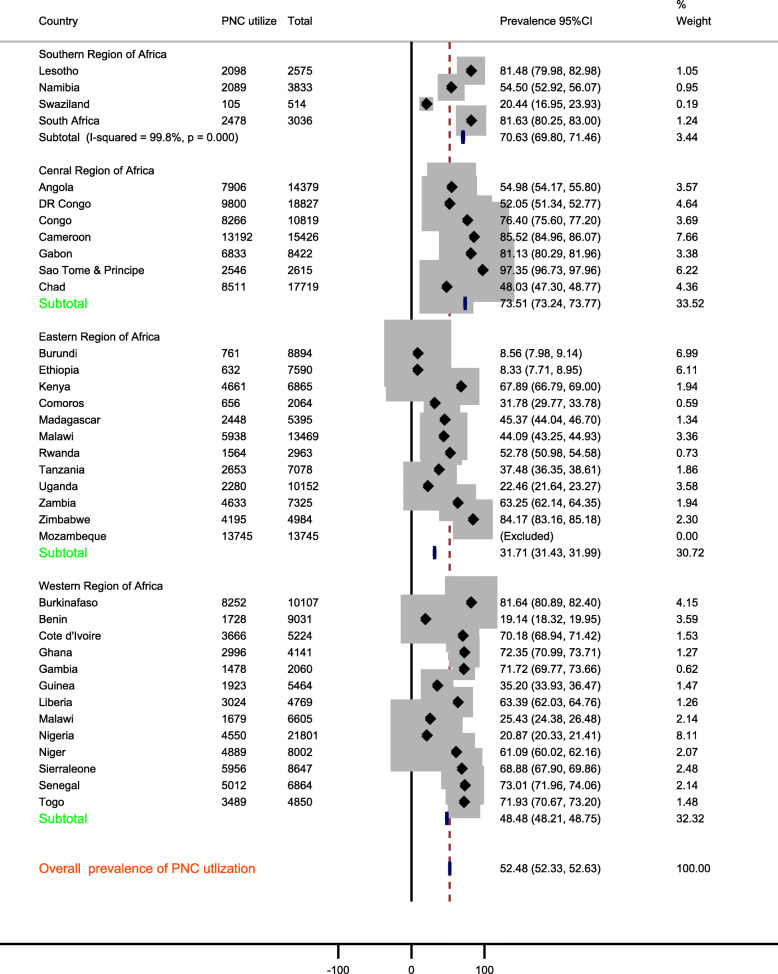
Map of study area

Ethiopia is found in East Africa of WHO region. It is located in the horn of Africa. Ethiopia had nine regions and two city administrations as shown in Fig. 1.

### Data source

We used data from the most recent Ethiopian Demographic and Health Survey 2016 conducted in January 18, 2016, to June 27, 2016. It is conducted every 5-year interval. The survey had different datasets (individual records, kids record, household record, men records, birth cohort records etc.). For this study, men’s record (MR) dataset was used. The data was freely accessible, and permission was obtained after projects are designed and submitted. The detail is found elsewhere [34].

### Sampling procedure

A two-stage stratified sampling procedure was adopted in selecting study participants. The detail of sampling procedure is found elsewhere [35]. All men aged 15–59 who had been interviewed about ever alcohol drinking were included in the study. However, respondents with missing data for the outcome variable were excluded. A total of 14,795 eligible male respondents were selected, and 12,594 were successfully interviewed, and the response rate was 85.12%.

### Variables of the study

#### Outcome variable

Respondents’ ever alcohol drinking status, the outcome variable in this study, was defined as a person who ever drinks alcohol in his lifetime.

#### Independent variables

The independent variables were grossly classified into the individual-level and community-level variables: Individual-level variable includes ever chewed khat, age, religion, marital status, educational status, sex of household head, working status, occupation, source of information (reading newspaper, reading magazines, and watching television), and wealth index (poor, middle and rich). The community-level variables include residence and region.

### Operational definition

Ever alcohol drinking was defined as a respondent who drinks alcohol during his lifetime.

Ever chat chewer was defined as a respondent who chewed chat during his lifetime.

### Data analysis procedure

To identify the predictors of alcohol use, the STATA 14 software was used. Sampling weight was done before any statistical analysis to adjust for the non-proportional allocation of the sample to different countries and the possible differences in response rates. Since the DHS data has hierarchical nature, measures of community variation/random-effects (intraclass correlation coefficient, median odds ratio [36], and proportional change in variance [37]) were estimated. The values of these measures were significant, indicating the use of a multilevel logistic regression model than ordinary logistic regression.

Model comparison was done using deviance between the null-model (a model with no independent variable), model I (a model with only individual-level factors), model II (a model with community-level factors), and model III (a model that contain both individual and community-level independent variables). A model with the lowest deviance (model III) was the best-fitted model.

Both bivariable and multivariable multilevel logistic regression were performed to identify the determinant factors of zinc utilization in Ethiopia. All variables with a *p* value < 0.25 at bivariable multilevel logistic model analysis were entered into the multivariable multilevel logistic regression model. *p* value ≤ 0.05 was used to declare statistically significant variables in the final model.

## Result

### Sociodemographic characteristics

Total weighted samples of 12,594 participants were included in the analysis. The median age of the respondent was 29 with an interquartile range (IQR) of 21–39. Almost half of the participants, 6426 (51.03%) were between the age of 15 and 29 years. The majority, 10,098 (80.18%), of the men were rural. The majority, 8154 (64.74 %), of them had media exposure. 5876 (46.66%) men were in the primary education class. Around two-third of 7705 (61.17 %) participants were married (Table 1).

**Table 1:** Socio-demographic characteristics of adult men 15–59 years in Ethiopia, EDHS 2016

Variables	Ever use alcohol		Total (%)
	Yes	No	
Ever chewed khat			
Yes	855	2562	3418 (72.86)
No	4157	5018	9176 (72.86)
Age group			
15–29	3640	2786	6426 (51.03)
30–44	2056	2116	4173 (33.13)
45–59	1030	9364	1995 (15.84)
Sex of the household head			
Male	5141	5893	11,034 (87.61)
Female	732	827	1560 (12.39)
Marital status			
Married	3726	3977	7704 (61.17)
Single	2147	2742	4890 (38.83)
Residence			
Urban	1415	1080	2495 (19.82)
Rural	4458	5640	10,098 (80.18)
Religion			
Orthodox	4939	732	5676 (45.07)
Muslim	345	3570	3916 (31.09)
Protestant	477	2267	2745 (21.80)
Others*	111	144	256 (2.03)
Education			
No education	1922	1846	3773 (29.96)
Primary	2452	3424	5876 (46.66)
Secondary	850	994	1845 (14.66)
Higher	643	455	1099 (8.73)
Region			
Tigray	723	11	794 (6.31)
Afar	8	73	82 (0.65)
Amhara	2679	527	3206 (25.46)
Oromia	1239	3474	4713 (37.43)
Somali	2	323	326 (2.59)
Benishangul	60	63	123 (0.98)
SNNPR	674	1911	2586 (20.53)
Gambela	19	17	36 (0.29)
Harari	5	26	31 (0.25)
Addis Ababa	441	179	620 (4.93)
Dire-Dawa	19	52	71 (0.57)
Media exposure			
Yes	4365	3788	8154 (64.74)
No	1508	2932	4440 (35.26)
Wealth index			
Poor	1754	2518	4272 (33.92)
Middle	1087	1339	2427 (19.27)
Rich	3031	2863	5895 (46.81)
Working status			
Yes	5266	5905	11,172 (88.71)
No	607	815	1422 (11.29)
Occupation			
Had occupation	5545	6105	16,650 (92.50)
Had no occupation	328	615	944(7.50)
Literacy			
Can read	4101	4476	8578 (68.11)
Cannot read	1772	2244	4016 (31.89)

### Random effect analysis

This study fits a model that considers the nature of the dataset. As known, the EDHS dataset had hierarchical nature. Therefore, fitting models that consider nature of the data is important. We fitted a generalized linear mixed effects model that had two component random effect and mixed effect. The fixed effect measures using odds ratio with the selected independent variables to qualify the effect size of low intake of food rich in vitamin A and independent variables. The random effect measures the variability of low intake of food rich in vitamin A. The variability measure for the random effect is community variance (1.34, *p* value < 0.001); intraclass correlation (ICC) (77.82%) indicates that there is intake of food rich in vitamin A at cluster level, median odds ratio (MOR) (3.83) means that if we randomly select children from different clusters, children at cluster of intake of food rich in vitamin A had 3.83 times higher odds of intake of food rich in vitamin A compare to its counterpart, and proportional change in variance (PCV) in model III was 88.38% the model best explains the variability of low intake of food rich in vitamin A. Model comparison was done using deviance. The lowest deviance (model III) was the best-fit model for this study (Table 2).

**Table 2:** Multilevel logistic regression analysis of both individual and community-level factors associated with alcohol use in Ethiopia, EDHS 2016

Individual and community-level variables	Models			
	Null model	Model I	Model II	Model III
	AOR (95%CI)	AOR (95% CI)	AOR (95% CI)	AOR (95% CI)
Men age				
15–29 years		1		1
30–44 years		1.28 (1.07, 1.54)		1.30 (1.08, 1.56)*
45–59 years		1.38 (1.09, 1.74)		1.38 (1.10, 1.74)*
Household head				
Male		1		1
Female		1.05 (0.87, 1.27)		1.09 (0.90, 1.32)
Marital status				
Had a partner		1		1
Not having a partner		0.78 (0.65, 0.94)		0.78 (0.64, 1.02)
Religion				
Orthodox		1		1
Muslim		0.006 (0.005,0.08)		0.01 (0.008, 0.044)
Protestant		0.05 (0.039, 0.062)		0.06 (0.054, 0.083)
Others		0.36 (0.24, 0.55)		0.42 (0.28, 0.63)
Men education				
Unable to read and write		1		1
Primary education		0.83 (0.65, 1.06)		0.87 (0.68, 1.11)
Secondary education		0.74 (0.55, 0.99)		0.80 (0.60, 1.08)
Higher education		1.30 (0.95, 1.78)		1.40 (0.99, 1.92)
Men working status				
Not working		1		1
Working		0.78 (0.54, 1.12)		0.83 (0.57, 1.02)
Media exposure				
No exposed		1		1
Exposed		1.67 (1.40, 2.00)		1.69 (1.41, 2.20)*
Wealth index				
Poor		1		1
Middle		0.91 (0.73, 1.13)		0.85 (0.68, 1.06)
Richer		0.84 (0.69, 1.03)		0.83 (0.68, 1.03)
Smoking status				
Non-smoker		1		1
Smoker		1.89 (1.48, 2.41)		2.18 (1.71, 2.79)*
Khat chewing				
No		1		1
Yes		2.77 (2.28, 3.37)		3.08 (2.54, 3.74)*
Occupation				
Had occupation		1		1
Had no occupation		0.30 (0.20, 0.46)		0.34 (0.22, 0.51)*
Literacy				
Can read		1		1
Cannot read		0.95 (0.76, 1.19)		1.01 (0.80, 1.26)
Residence				
Urban			1	1
Rural			0.27 (0.17, 0.41)	0.80 (0.56, 1.15)
Region				
Tigray			1	1
Afar			.0007 (.0003, 0.0007)	0.02 (0.009, 0.042)*
Amhara			0.62 (0.31, 1.25)	1.05 (0.61, 1.81)
Oromia			0.008 (0.004, 0.017)	0.06 (0.039, 0.11)*
Somali			0.0001 (0.0003, 0.00)	0.004 (0.001, 0.011)*
Benishangul			0.04 (0.021, 0.093)	0.33 (0.18, 0.58)*
SNNPR			0.1 (0.005, 0.02)	0.07 (0.04, 0.12)*
Gambela			0.03 (0.016, 0.073)	0.15 (0.08, 0.27)*
Harari			0.02 (0.001, 0.005)	0.01 (0.008, 0.032)*
Addis Ababa			0.048 (0.02, 0.106)	0.21 (0.11, 0.39)*
Dire Dawa			0.003 (0.001, 0.007)	0.03 (0.016, 0.059)*
**Random effects**				
**ICC%**	**77.82**	**47.23**	**48.84**	**29.04**
**PCV%**	**1**	**74.52**	**72.79**	**88.38**
**MOR**	**51.41**	**7.33**	**7.83**	**3.83**
**Model fitness**				
**Log-likelihood ratio**	**− 5224**	**− 3920**	**− 4907**	**− 3690**
**Deviance**	**10448**	**7840**	**9817**	**7380**

*CI* confidence interval, *AOR* adjusted odds ratio, *Others* traditional religion followers

*Significant at *p* value = 0.05

### Fixed effects analysis result

The age group has a significant effect on alcohol drinking. The odds of alcohol drinking when compared with people 15–20 years of age or older (20–34, 35–49) increase by 30% and 38% (AOR = 1.30, 95% CI 1.08, 1.56 and AOR = 1.38, 95% CI 1.10, 1.74). Being Muslim, Protestant, and other religion follower decreases the odds of alcohol drinking 99.4%, 95%, and 64% (AOR = 0.006, 95% CI 0.005, 0.008; AOR = 0.05, 95% CI 0.039, 0.062; and AOR = 0.36, 95% CI 0.24, 0.55) as compared to Orthodox religion followers respectively. Media exposure had a relationship with alcohol drinking. The odds of alcohol drinking among media-exposed men increase by 67% as compared to non-exposed men (AOR = 1.67, 95% CI 1.41, 2.20). Smoking had a significant effect on alcohol drinking. The odds of alcohol drinking among smokers were 2.18 times higher risk as compared to non-smokers (AOR = 2.18; 95% CI 1.41, 2.20). There is a strong relationship between khat chewing and alcohol drinking. The odds of alcohol drinking among khat chewers were 3.08 times higher as compared to non-khat chewers (AOR = 3.08, 95% CI 2.54, 3.74). There is a relationship between the occupation of men and alcohol drinking. The odds of alcohol drinking among men who had occupation decrease by 66% as compared to men who had no occupation (AOR = 0.34, 95% CI 0.22, 0.51). The odds of alcohol drinking among men who live other than in Tigray decreases (Table 2).

## Discussion

This study showed the prevalence and associated factors of alcohol use in Ethiopia using the Ethiopian Demographic and Health Survey of 2016. The prevalence of lifetime alcohol use was 46.64% (95% CI 45–47%). The current finding was significantly lower than other findings in New Zealand [5], in India [14], in Nigeria [15], in Uganda [16], and in Nigeria [17]. The discrepancy might be due to that in New Zealand the age was 16–64 years, and the survey was a cohort study. In India, the study was conducted on only rural area dwellers, who are a highly vulnerable population for alcohol use, which is supported by other findings [24]. In Nigeria, researches were conducted in semirural dwellers and militaries who were more prevalent in the alcohol use population. In Sri Lanka, the study participants were mentally ill individuals.

When compared with people 15–20 years of age or older (20–34, 35–49), individuals have a higher risk alcohol use. This finding is consistent with other studies that have found an association of alcohol use with increasing age [24] and of being aged 30 and over [25, 26]. Possible reasons include older people are independent of family control, and they have also their own income generation.

Muslim, Protestant, and other religion followers decrease the odds of alcohol drinking 99.4%, 95%, and 64% as compared to Orthodox religion followers. The finding is consistent with other findings (51.6%) [15, 20]. The reason might be Orthodox followers are culturally accepted in Ethiopia to drink alcohol.

In this study, the odds of alcohol use was about 1.69 [AOR = 1.69; 95% CI (1.14, 2.20)] times higher than those who were following media as compared to individuals who were not following media. There is evidence that alcohol consumption increase in Ethiopia from time to time [22]. Currently in Ethiopia, alcohol advertising though media is stopped by the Ethiopian government [31].

The study participants who smoked tobacco and chewed khat were highly significant with alcohol use when compared with their counterparts. This finding is supported by other findings [20, 23, 24]. The reason could be the action of alcohol is sedation [38]; to break the sedation, alcohol user also uses stimulants like khat and cigarette to get the feeling of being energized and hyperalert [39].

The odds of alcohol drinking among men who had occupations decreased by 66% as compared to men who had no occupation. This finding is supported by studies elsewhere [40]. Unemployment is an important factor for alcohol use, and problematic alcohol use crimp the likelihood of unemployment and decreases the chance of finding and holding down a job [41–43]. In Ethiopia, the government has no control over the production of locally brewed alcoholic drinks. Therefore, alcoholic beverage is found everywhere and everybody can access at low cost [44]. Unemployed men have more time available during the day in which to drink alcohol.

## Conclusion

Nearly half of the Ethiopian population reported alcohol use at least once in their lifetime. Old age, being Orthodox religion followers, media exposure, khat chewing, smoking, and having no occupation were predictors of alcohol use in Ethiopia. Therefore, health education about the risk of alcohol used is highly recommended. In addition, khat chewing and smoking control mechanisms should be designed and given spatial attention. Job opportunities should be also created for the majority of society to alleviate alcohol use in Ethiopia.

## Limitation of the study

Since the study is cross-sectional, it is not possible to establish a causal relationship between the independent and dependent variables. The study did not look at the pattern (frequency, dose) of alcohol use, harmful drinking, clinical aspects and consequences of dependence, and the consequence. The outcome measure for this study was by asking questions not by blood chemistry confirmation, and this may affect this study result.

## Data Availability

The datasets used during the current study are available from the corresponding author.

## Abbreviations

AOR: Adjusted odds ratio
CI: Confidence
LLR: Likelihood ratio
RR: Relative risk
SNNPR: South Nation Nationalities of People Regions
EDHS: Ethiopian Demographic and Health survey
