# Correction to: Prevalence and predictors of alcohol use among adult males in Ethiopia: multilevel analysis of Ethiopian Demographic and Health Survey 2016

**DOI:** 10.1186/s41182-020-00290-z

**Published:** 2020-12-22

**Authors:** Zemenu Tadesse Tessema, Tadele Amare Zeleke

**Affiliations:** 1 Department of Epidemiology and Biostatistics, Institute of Public Health, College of Medicine and Health Science, University of Gondar, Gondar, Ethiopia; 2 Department of Psychiatry School of Medicine, College of Medicine and Health Science, University of Gondar, Gondar, Ethiopia

**Correction to: Trop Med Health 48, 100 (2020)**

**https://doi.org/10.1186/s41182-020-00287-8**

Following the publication of the original article [1], the authors identified an error in Fig. 1. The correct figure is given below.

**Fig. 1:**
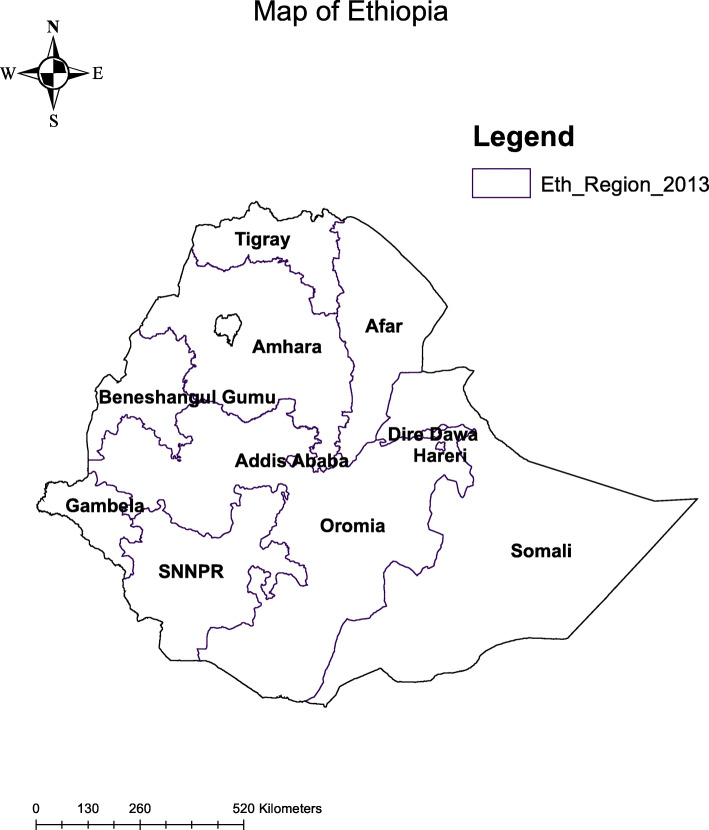
Map of study area

The original article has been corrected.
